# Development of a New Sensitive Method for Lead Determination by Platinum-Coated Tungsten-Coil Hydride Generation Atomic Absorption Spectrometry

**DOI:** 10.1021/acsomega.3c01856

**Published:** 2023-06-09

**Authors:** Muhammet Atasoy

**Affiliations:** Muğla Vocational School, Chemistry and Chemical Treatment Technologies Department, Chemistry Technology Program, Muğla Sıtkı Koçman University, 48000 Muğla, Turkey

## Abstract

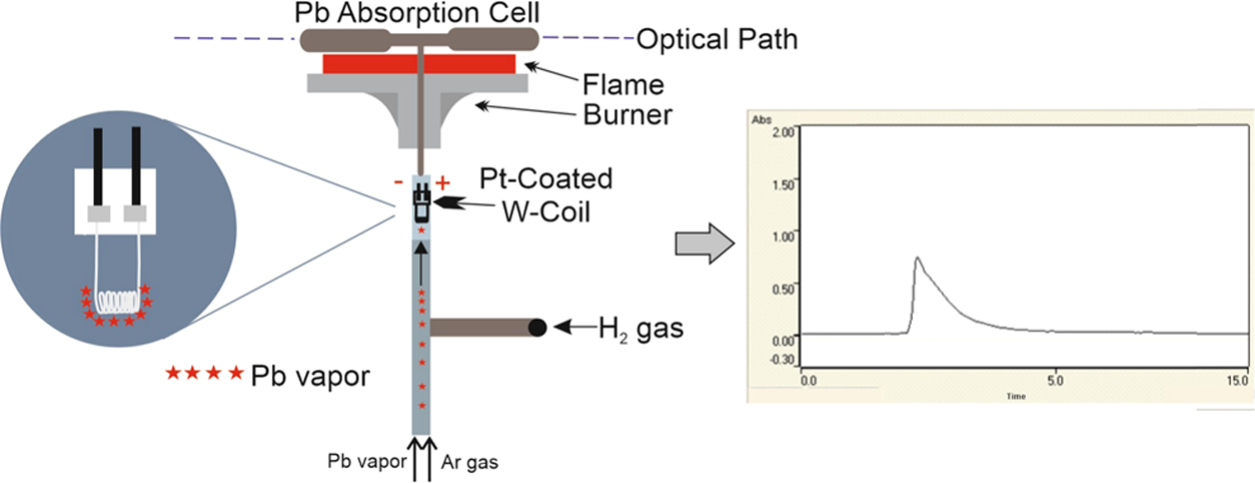

A novel very sensitive and rapid analytical method was improved where gaseous lead formed was transported to and trapped on an externally heated platinum-coated tungsten-coil atom trap for in situ preconcentration. The analytical performance of the developed method with the graphite furnace atomic absorption spectrometry (GFAAS) method was compared. All critical parameters affecting the performance of both methods were optimized. The limit of quantitation (LOQ) was found as 11.0 ng L^–1^ and the precision was 2.3% in terms of percent relative standard deviation (RSD%). Characteristic concentration (Co) of the developed trap method was indicating a 32.5-fold enhancement in sensitivity compared to the GFAAS method. In order to investigate the surface morphology of the W-coil, scanning electron microscope–energy-dispersive X-ray (SEM-EDS) analyzes were performed. The accuracy of the trap method was tested by certified reference materials: NIST SRM 1640a (the elements in natural water) and DOLT:5 (dogfish liver). Interferences from other hydride-forming elements were investigated. Application of the trap method was demonstrated by the analysis of some drinking water and fish tissue samples. The t test was applied to drinking water samples, and the results indicated that there was no statistically significant error.

## 1 Introduction

Lead (Pb) is one of the most harmful toxic elements causing serious health problems.^1^ Exposure of antioxidants and enzymes in cells to Pb results in increased reactive oxygen species that lead to numerous dysfunctions in DNA, lipids, and proteins.^2^ Furthermore, it has the potential to induce encephalopathy, cognitive dysfunction, renal damage, anemia, and neurologic toxicity.^3^ Pb is inevitably released into the environment through human activities, such as pollution from industrial production and heavy traffic activities.^4^ Although countries are constantly tightening their environmental regulations and developing waste management technologies in parallel, Pb pollution will continue to be a major problem as a large amount of Pb still circulates in soil and water.^5^ The presence of these toxic elements in water resources threatens public health. Nowadays, access to clean water resources has become a global challenge.^6^ The World Health Organization (WHO) has set the highest allowed Pb concentration in drinking water as 10.0 μg L^–1^.^7^ Humans can also be exposed to toxic elements through diet. Including fish in the diet is considered a healthy choice due to its high nutritional value, which includes high-quality proteins, vitamins, omega-3 fatty acids, and minerals.^8^ It is also a fact that fishes live in aquatic environments containing many toxic elements. Fishes are often used as a biological indicator for heavy-metal pollution in water systems.^9^ Frequent consumption of fish and fishery products can lead to the accumulation of trace metals such as Pb, even at low concentrations, which can cause significant health problems in humans. There is current worldwide concern about the detection of toxic elements in fish.^10^ Hence, the development of novel, rapid, and robust techniques to precisely and accurately quantify the level of Pb in environmental and biological samples is of the greatest importance.^3^

To determine the level of Pb in different matrixes, various analytical methods have been utilized, including electrochemistry,^11^ inductively coupled plasma mass spectrometry (ICPMS),^12^ inductively coupled plasma optical emission spectrometry (ICPOES),^13^ hydride generation atomic fluorescence spectrometry (HGAFS),^14^ graphite furnace atomic absorption spectrometry (GFAAS),^15^ and hydride generation atomic absorption spectrometry (HGAAS).^16^ Among these methods, GFAAS and ICPMS are the most commonly used in laboratories. However, GFAAS may encounter problems such as linear range, analytical sensitivity, and matrix interference.^17^ In addition, the high cost of graphite tubes limits the use of this method. The instrument used in the ICPMS method is very expensive and the operating cost is very high for only mono elemental analysis.^18^ Besides these methods, the determination of elements that form hydrides is typically accomplished using HGAAS, a well-established analytical technique.^19^ Hydride generation offers significant advantages in terms of more efficient transport and excitation of gaseous-formed analytes to the atomization source.^12^ The interferences in AAS can be broadly categorized as spectral and nonspectral. Spectral interferences arise due to radiation absorbed by species other than nonanalyte atoms, whereas nonspectral interferences are caused by the effect of other species in the sample matrix on the analyte of interest. In the HGAAS method, spectral interferences are not of much concern as it efficiently separates the analyte from the matrix. Lead hydride (PbH_4_) generation is difficult due to its low yield and stability. Various reagents have been used to increase the efficiency of hydride generation.^19^ These reagents are used for the oxidation of the unstable form Pb(II) to the hydride-forming form Pb(IV).^12^ In previous studies, among the reagents of dichromate, permanganate, cerium (IV), hydrogen peroxide, peroxide disulfate, and potassium hexacyanoferrate (III) used as oxidation agents, it was stated that potassium hexacyanoferrate (III) gave the highest sensitivity in the hydride generation medium.^19^

Researchers have developed some trap methods to achieve very low detection limits.^20^ Moreover, the better sensitivity of the trap system leads to greater dilution of sample constituents, thereby decreasing the effect of diluted interferents on the analyte under study.^21^ In previous studies, it was reported that interference effects can be significantly eliminated by changing the trap temperature.^22^ The trap of hydride-forming elements in a graphite furnace (GF) is one of the most common methods used for hydride trap, but the obtained limit of detection values in the lead determination are lower than the other hydride-forming elements. Therefore, lead trapping in GF could not gain enough popularity.^16^ In some studies in the literature, a quartz surface was used for atom trapping. Kratzer^16^ both trapped and atomized PbH_4_ on the quartz tube surface and determined Pb at an ultratrace level. In another study by Uslu et al.,^23^ Pb was determined at the ng L^–1^ level by conventional AAS using a T-shaped slotted quartz tube trap. In many studies, analyte atoms were trapped on the surface of a W-coil. In a study by Cankur and Ataman,^24^ the application of a resistively heated W-coil surface led to the successful trapping and revolatilization of Cd atoms. In another study, Alp and Ertaş^25^ in situ trapped arsenic hydrides on the W-coil surface by HGAAS. In the aforementioned study, it was coated with iridium, resulting in a significant reduction in interference effects. The coating of its surface with appropriate elements enabled the selective and sensitive determination of analyte atoms. In a study conducted by Liu et al.,^26^ its surface was coated with different noble elements. Bismuthine was on-line trapped on coated W-coil and then electrothermally vaporized for determination by AFS. The study revealed that the Ir-coated W-coil performed the best. Yildiz et al.^21^ determined ultratrace levels of arsenic in drinking water samples using the HGAAS method after coating the W-coil surface with platinum. In addition, Atasoy and Kula^27^ proposed a new technique for selenium determination and speciation by coating the W-coil surface with gold and combining it with the HGAAS method.

This study aims to develop a highly sensitive, fast, simple, robust, and cost-effective method for the determination of Pb in some drinking water and fish tissue samples. Pt-coated W-coil is used as an on-line trap after lead hydride generation before atomization in the quartz absorption cell. To the best of my knowledge, this study is the first to demonstrate the trapping, preconcentration, and revolatilization of Pb using a Pt-coated W-coil atom trap HGAAS method. All experimental parameters were optimized. The analytical characteristics of the developed method were compared with the GFAAS method. Interferences of hydride-forming elements were investigated in detail. Finally, the applicability of the method to real samples was demonstrated.

## 2 Experimental Section

### 2.1 Reagents

All reagents used in experimental studies were analytical reagent grade or higher purity and all reagents were supplied by Merck (Darmstadt, Germany). All working solutions were prepared in deionized water (Millipore, 18.2 MΩ·cm). Argon (Ar) gas purity of 99.999% was employed as the carrier gas that transports the hydride products from the gas–liquid separator to the nebulizer/burner unit. The H_2_ gas used in the trap experiments was also of high purity (99.999%). Compressed medical purity acetylene was used as the source of air–acetylene flame. Working Pb standard solutions were prepared fresh daily by diluting 1000 mg L^–1^ Pb stock solution in K_3_[Fe(CN)_6_]. NaBH_4_ was prepared daily and stabilized with NaOH (Suprapur) solution to decrease its rate of decomposition. HCl (Suprapur) was used as the acid medium for the trap studies. In order to test the accuracy of the trap approach, DOLT:5 dogfish liver (National Research Council Canada) and NIST SRM 1640a trace elements in natural water (National Institute of Standards & Technology) certified reference materials were used. Standard solutions of the elements investigated for interference effects, namely, Hg, Sb, Sn, Bi, As, and Se, were prepared by diluting their 1000 mg L^–1^ stock solutions.

### 2.2 Apparatus

For the determination of lead in the first part of this study, an Agilent Technologies GTA 120 graphite furnace atomic absorption spectrometer equipped with a Zeeman background technique and PSD120 autosampler was used. The Pb hollow cathode lamp was operated at 10.0 mA, the spectral bandpass was set to 0.5 nm, and the wavelength was set to 283.3 nm. To conduct trap studies, an Agilent 240 FS atomic absorption spectrometer equipped with a VGA 77 hydride generator system was employed. The analytical measurements were corrected for background using a deuterium system. The VGA 77 hydride generator had separate flow-rate settings for a sample and reducing agent/acid that could be changed by tightening or loosening the adjustment knob. The Pb hollow cathode lamp was operated at 5.0 mA, the spectral bandpass was set to 1.0 nm, and the wavelength was set to 217.0 nm. The absorbance measurements of Pb are based on the peak area for the GFAAS method and the trap studies are based on the peak height.

The quartz T-tube atomizer was placed on the burner by a standard cell holder and heated externally with an air–acetylene flame. It had a horizontal arm with dimensions of 140 mm in length, 18 mm in outer diameter (o.d.), and 15 mm in inner diameter (i.d.). The vertical arm was 100.0 mm in length, 9.0 mm in o.d., and 6.0 mm in i.d. A smaller quartz tube, 140.0 mm in length, was attached to the end of the vertical arm, and a hole was drilled in the middle of this tube to accommodate the tungsten coil (W-coil) obtained from a projector bulb (OSRAM, Germany). The W-coil was placed inside the quartz tube, with the electrical terminals of the coil on the outside and the coil portion inside the tube, using a flame-retardant and leak-proof stove band made of aluminum to facilitate coil replacement when necessary. A black fluoroelastomer tubing was used to connect the vapor outlet of the gas/liquid separator to the inlet stem of the tube, and its length was kept as short as possible for good analytical practice. The trap temperature was provided by a power supply (TT T-ECHNI-C, China) that can be manually adjusted. The corresponding temperature values to the current values were obtained using a thermocouple (Testo 925, Germany).

### 2.3 Surface Treatment Procedure

The procedure of coating was achieved by manually pipetting a 20.0 μL aliquot of 1000 mg L^–1^ Pt solution in 10% HCl onto the W-coil surface. It was then exposed to a heating protocol consisting of 3.8 A for 60 s, 4.2 A for 30 s, 0 A for 5 s, and 7.0 A for 5 s, which was replicated several times. Throughout the surface treatment procedure, the H_2_ and Ar gas flow rates were held constant at 40.0 mL min^–1^ and 300.0 mL min^–1^, respectively, as reported by Yildiz.^28^

### 2.4 General Procedure

Experimental studies were carried out for both the GFAAS method and trap approach in the scope of this study. The GFAAS method was used as a reference to compare the performance of the trap method. Experimental parameters that are important for both methods were optimized. During the optimization studies, a univariate optimization was implemented. While changing the value of the investigated parameter, the others were kept constant. The optimization of experimental parameters was first performed for the HGAAS method. In this method, the furnace program was optimized. As a matrix modifier, 1000 mg L^–1^ Pd solutions are used.^18^ 10.0 μL of this solution was injected into Pb standard solutions. Optimization studies were performed using 10.0 μg L^–1^ Pb solutions.

**Figure 1:**
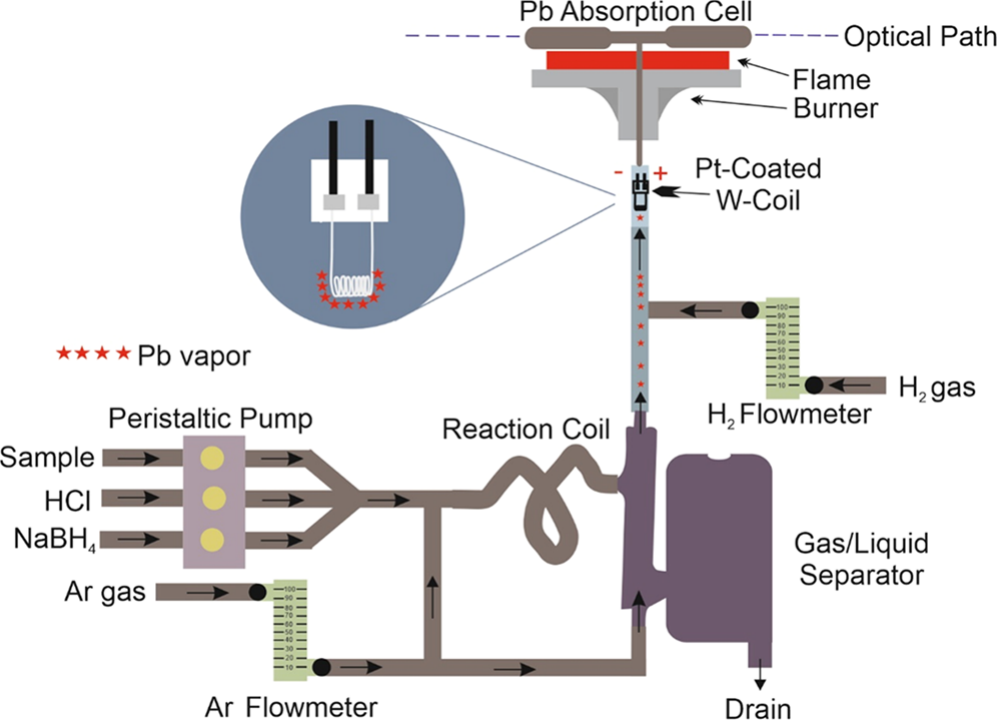
Schematic diagram of the trap method.

Optimization studies were then conducted for the trap method. The trap procedure used in this study consists of two steps: trapping and releasing. Ar and H_2_ gases were introduced to the trap system in both steps and the flow rates were controlled using flow meters. The connection of H_2_ gas was done close to the W-coil and sent to the trap system. The aim here is to obtain sharper analytical signals by allowing the H_2_ gas to reach the trap system in a very short time. In the trap step, a very small amount of H_2_ gas was introduced into the trap system first. The power supply was used to reach the optimal temperature for the trap, after which the peristaltic pump of the VGA 77 hydride system was activated. The acid, sample solution, and reducing agent were sent to the system through separate tubing. Pb vapor was trapped on the surface of the Pt-coated W-coil for a certain period. In the releasing step, the peristaltic pump was first turned off. Then, the H_2_ gas was simultaneously increased to the optimal flow rate and the trap temperature was adjusted to the optimal releasing temperature. After a few seconds, the H_2_ gas supplied to the system was stopped and the power supply was turned off. During this time, the highest level of volatility efficiency was attained, accompanied by the detection of a transient signal. The experimental setup of the trap system is presented in Figure 1.

### 2.5 Sample Pretreatment

Each drinking water sample obtained from the Muğla Province was acidified to contain 1.0 mol L^–1^ HNO_3_ and stored in a refrigerator until analysis. Before analysis, K_3_[Fe(CN)_6_] was added to each of them. To compare the results obtained, a *t* test was performed at a 95% confidence level using Microsoft Excel. In this test, *t*_calculated_ (calculated from the sample data) is compared with *t*_critical_.^29^ This value is based on the *t*-distribution read from the table. Two fully grown fish species purchased from a local market in the Muğla Province of Turkey were among the most preferred fish types for the diet of the local population. The liver, muscle, and gill tissues of each fish sample were dissected using a sterilized scalpel. Approximately 0.1–0.5 g of samples was weighed and placed in Teflon vessels, and 10 mL of 70% (w/w) HNO_3_ was added. Microwave digestion was employed using the CEM Mars 6 system to digest the samples. The operating conditions for fish tissue samples were carried out by applying the same procedure suggested by Atasoy et al.^20^ Fish tissue samples were subjected to digestion using the food program. The temperature was gradually raised to 210 °C over 20 min and held constant at this level for 15 min. After cooling down, the digested samples were diluted with ultrapure water to a final volume of 50 mL. For DOLT:5, three different weights of 0.1 g each were taken and transferred to Teflon vessels in the microwave digestion unit. All of the pretreatments applied to the fish samples were also applied to this certified reference material samples, and they were digested using the same digestion procedure.

## 3 Results and Discussion

### 3.1 Optimization Studies Carried Out in the GFAAS Method

The parameters in the GFAAS method were optimized to enhance the analytical signal of Pb. 10.0 μL of Pd solution (1000 mg L^–1^) was injected into Pb standard solutions as a modifier. The chemical matrix of the analyte is important in determining the optimum ashing and atomization conditions. The atomization temperature can be altered by using chemical modifiers such as palladium, allowing for a higher ashing temperature to be achieved.^30^ The Ar flow rate was 300.0 mL min^–1^. All measurements were performed using integrated absorbance (peak area). Pyrolysis/atomization temperatures were optimized.^31^ The optimum furnace program is given in Table 1.

**Table 1:** Optimum Furnace Program of GFAAS

step	temperature (°C)	time (s)	Ar flow rate (L min^–1^)
dry			
1	85	5	0.3
2	95	40	0.3
3	120	10	0.3
pyrolysis			
1	400	5	0.3
2	400	1	0.3
3	400	2	0.0
atomization			
1	2100	1	0.0
2	2100	2	0.0
3	2100	2	0.3

### 3.2 Optimization Studies in the Pt-Coated HGAAS Method

**Figure 2:**
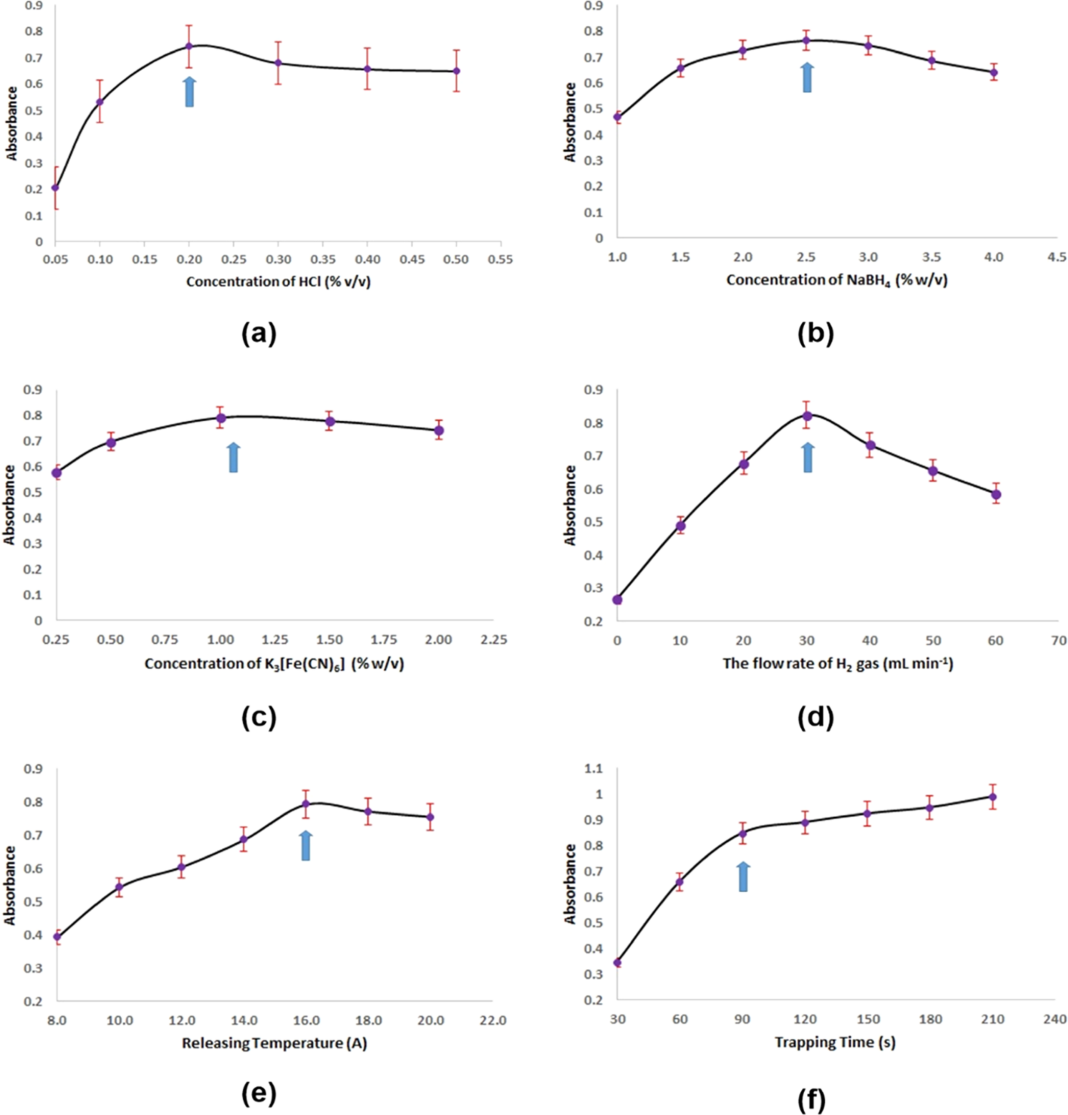
(a) Effect of HCl concentration on the Pb signal. (b) Effect of NaBH_4_ concentration on the Pb signal. (c) Effect of K_3_[Fe(CN)_6_] concentration on the Pb signal. (d) In the trapping step, the effect of H_2_ flow rate on the Pb signal. (e) Effect of releasing temperature on the Pb signal. (f) Effect of trapping time on the Pb signal.

Optimized experimental parameters are concentrations of NaBH_4_, NaOH, HCl, and K_3_[Fe(CN)_6_] solutions, trapping time, trapping and releasing temperatures, and flow rates of Ar and H_2_ gases. While determining the optimum value of a parameter, reproducible and stable signals were taken as the basis for the optimum value of the investigated parameter. Optimization studies were carried out using 2.5 μg L^–1^ Pb solutions prepared in K_3_[Fe(CN)_6_]. First, the optimum concentration of the solutions used in the trap method was determined. The concentration of the HCl solution was varied between 0.05 and 0.5 (v/v) mol L^–1^, and the optimum value was found to be 0.2 mol L^–1^ (v/v), as shown in Figure 2a. Gradual decreases in the analytical signal of Pb occurred at higher values. NaBH_4_ solutions were prepared at concentrations ranging from 0.1 to 4.0% (w/v), and the effect on the analytical signal of the reducing reagent was investigated. Very low absorbances were obtained at concentrations below 1.0% (w/v). As shown in Figure 2b, it was found that the optimum concentration of NaBH_4_ was 2.5% (w/v). On the other hand, the study on optimizing NaOH concentration utilized solutions with concentrations ranging from 0.1 to 0.6% (w/v), with the best concentration of NaOH determined to be 0.3% (w/v). The NaOH concentration did not appreciably change the analytical signal. The signals obtained when K_3_[Fe(CN)_6_] was not added to Pb solutions were both unstable and very low. The optimal concentration of K_3_[Fe(CN)_6_] in Pb solution was determined by varying the concentration from 0.25 to 2.0% (w/v). Five different K_3_[Fe(CN)_6_] powders ranging from 0.25 to 2.0 g were weighed and transferred to 100 mL volumetric flasks. To each flask, a Pb standard solution was added to a final concentration of 2.5 μg L^–1^, and the volumes were made up to 100 mL with ultrapure water. The results depicted in Figure 2c indicated that the optimal concentration of K_3_[Fe(CN)_6_] was 1.0% (w/v). The flow rate of the sample solution was different from that of the reducing and acid solutions. Specifically, the flow rates of the reducing or acid solutions and the sample solutions were 4.95 and 4.55 mL min^–1^, respectively.

Among the critical parameters investigated in the trap study, the flow rate of the H_2_ gas was one of the most important. In both the trapping and releasing steps, the optimal flow rate of H_2_ gas was determined. By introducing H_2_ gas during both steps, the atom trap could be protected from oxidation and the revolatilization of trapped lead vapor species could be enhanced. In the absence of H_2_ gas during the trapping step, the analytical signals were low. However, introducing even small amounts of H_2_ gas significantly increased the analytical signal of Pb. When the flow rate of H_2_ gas was increased to 30.0 mL min^–1^, the analytical signal increased gradually, but above this value, the signal started to decrease. It was also observed that the trap temperature decreased as a result of high H_2_ gas amounts during the trapping step. As shown in Figure 2d, the optimum value of the H_2_ gas was determined as 30.0 mL min^–1^ for the trapping step. The amount of H_2_ gas introduced to the system during the releasing step was rapidly increased simultaneously with the trap temperature. When an insufficient amount of H_2_ gas was introduced to the system in this step, unstable and splayed signals were obtained. When excessive amounts of H_2_ gas were introduced, sharp signals were observed, but the obtained analytical signals were low. The optimum value of H_2_ gas introduced to the system for the releasing step was found to be 140.0 mL min^–1^. Ar gas sent to the trap system in both steps was kept constant and the optimum flow rate was determined as 208 mL min^–1^.

Other most important parameters are the trapping and releasing temperatures. The temperature of the trap was increased using a power supply. While the optimum values of the trapping and releasing temperatures were determined, the current values applied to the trap were increased gradually. Then, the temperature values corresponding to the current values under optimum experimental conditions were determined with the thermocouple. It was found that a small amount of analyte atoms were trapped on the trap surface when no external temperature was applied to the trap system. However, with each increase in temperature, the analytical signal displayed a gradual increase. The signals began to decrease at trapping temperatures above 85 °C (2.0 A). It is thought that as the applied temperature increases, the collected analyte atoms on the trap are also detached from the trap surface. The same trap was used throughout all studies and no loss of sensitivity was observed. This also proves that the Pt-coated W-coil is highly resistant to heat. As shown in Figure 2e, the optimal releasing temperature was found to be 940 °C (16.0 A).

At trapping times of less than 90 s, very low analytical signals were obtained. On the other hand, increases in analytical signals were observed at trapping times over 90 s. It is believed that there is enough active surface area on the trap surface to trap more analyte atoms, and the reason for this increase was attributed to it. It was concluded that 90 s was sufficient for the optimum trapping time, as shown in Figure 2f. As the trapping time increases, so does the consumption of H_2_ and Ar gases. In addition, it causes waste of solutions such as HCl and NaBH_4_, which means that the cost of the developed method increases. The optimal values of the experimental parameters in the trap method are summarized in Table 2.

**Table 2:** Optimized Parameters for the Trap Method

analytical parameters	optimum values
carrier solution	0.2 mol L^–1^ HCl, 4.95 mL min^–1^
reductant solution	2.5% (w/v) NaBH_4_, stabilized in 0.3% (w/v) NaOH, 4.95 mL min^–1^
sample solution	2.5 μg L^–1^ Pb stabilized in 1.0% (w/v) K_3_[Fe(CN)_6_], 4.55 mL min^–1^
carrier gas in the trapping step	208 mL min^–1^ Ar; 30 mL min^–1^ H_2_
carrier gases in the releasing step	208 mL min^–1^ Ar; 140 mL min^–1^ H_2_
trapping temperature	85 °C
releasing temperature	940 °C
trapping time	90 s

**Figure 3:**
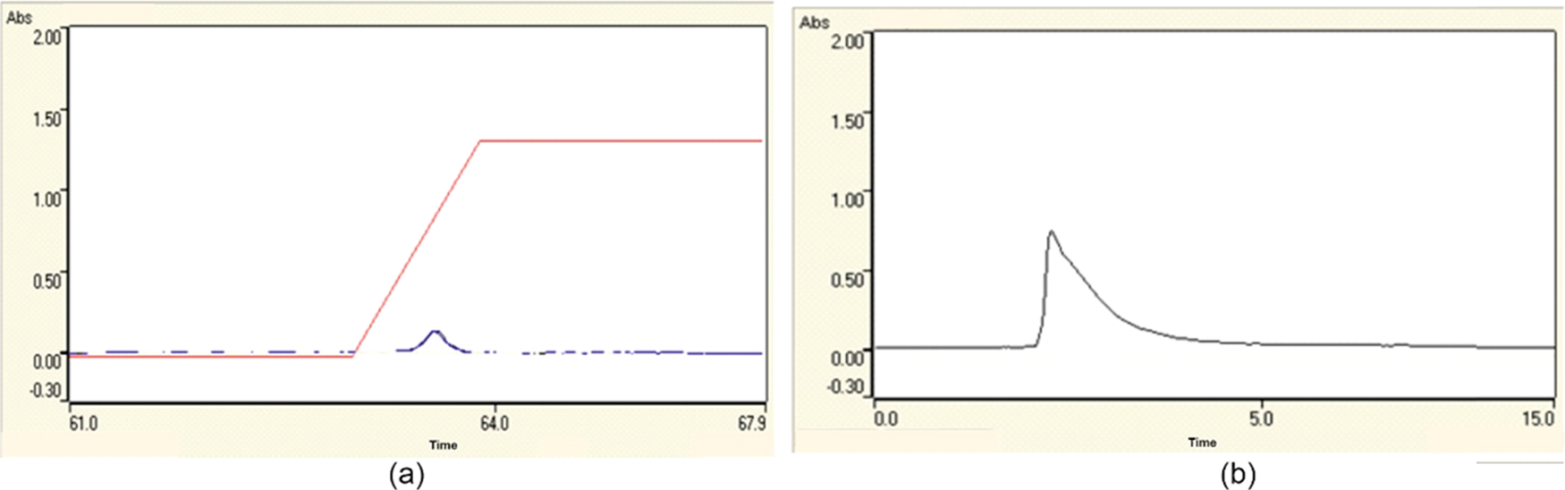
(a) Signal of 20.0 μg L^–1^ Pb obtained by the GFAAS method. (b) Signal of 2.5 μg L^–1^ Pb obtained by the trap method (90 s trapping time; 6.83 mL sample volume).

The analytical signal of the 20.0 μg L^–1^ Pb using the GFAAS method is given in Figure 3a and the analytical signal of the 2.5 μg L^–1^ Pb using the trap method obtained for 90 s trapping time is also given in Figure 3b.

### 3.3 SEM-EDS Results

**Figure 4:**
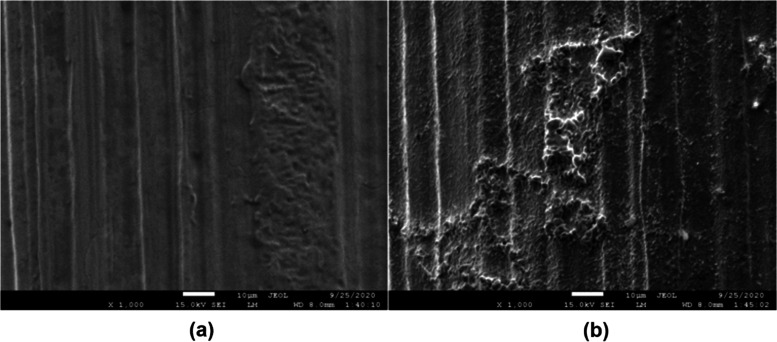
SEM images of (a) bare W-coil and (b) Pt-coated W-coil.

A scanning electron microscope with energy-dispersive spectroscopy (SEM-EDS) (JEOL JSM-7600F instrument from Mugla Sitki Kocman University) was used to examine the W-coil surface morphology. Figure 4 shows SEM images of the bare W-coil and Pt-coated W-coil at a magnification of 1000.

**Figure 5:**
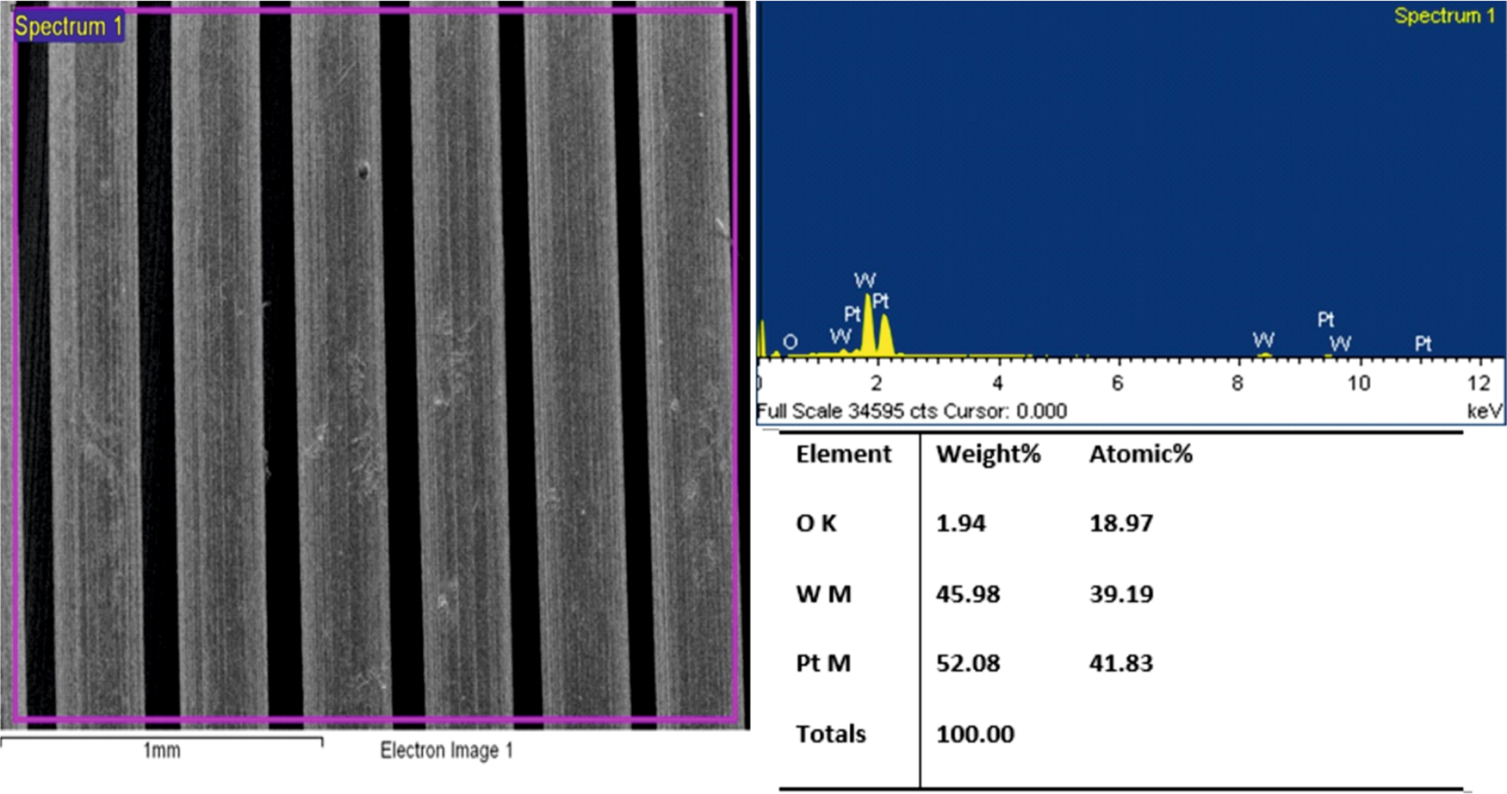
EDS result of Pt-coated W-coil.

As seen in Figure 4b, the thickness of the coating showed surface morphology with higher roughness. With the coating of Pt, some of the roughness on the W-coil surface has been adjusted. This has caused surface defects with cracks, voids, or other imperfections. The roughness on the surface was not homogeneously distributed, but this result did not adversely affect the efficiency of the coating and the results of the experimental study. As can be seen from the EDS result provided in Figure 5, elemental Pt is distributed on the surface of the W-coil at a weight percentage of 52.08%. This coating percentage is sufficient to trap the volatile lead vapor sent to the trapping system.

### 3.4 Analytical Features

Table 3 presents the analytical performance resulting from the application of both the developed trap method and the GFAAS method. Calibration pilots were established using the optimum values of the experimental parameters, and linearity ranges were determined. The peak area values were taken as a basis for the GFAAS method. The linear calibration range of the GFAAS method covered Pb concentrations ranging from 5.0 to 200.0 μg L^–1^, with a correlation coefficient (*R*^2^) of 0.9958. When calculating the limit of detection (LOD) and limit of quantitation (LOQ) values, first, 11 times blank solution absorbance measurements were obtained, and then the standard deviation of these values was calculated. The LOD value was calculated by dividing the three-fold of this standard deviation value by the slope of the calibration curve. The LOQ value was calculated by dividing the 10-fold of the same standard deviation value by the slope of the calibration curve. For the GFAAS method, LOD (3 s) and LOQ (10 s) values were obtained to be 0.217 and 0.724 μg L^–1^, respectively. The precision, RSD% was determined to be 3.5%.

**Table 3:** Analytical Response Characteristics of the Trap Method and the GFAAS Method

	Pt-coated W-coil HGAAS	GFAAS
LOD, ng L^–1^	3.3	217.3
LOQ, ng L^–1^	11.0	724.4
RSD% (*n* = 11)	2.3	3.5
linear range, μg L^–1^	0.01–10.0	5.0–200.0
*C*_0_, ng L^–1^	21.3	691.8
calibration equation^a^	*y* = 0.2029[Pb] + 0.0344	*y* = 0.0058[Pb] + 0.0221
sample volume, mL	6.83	0.01
trapping time, s	90	

a *y* is absorbance and [Pb] is concentration of Pb in μg L^–1^.

During the trap experiments, peak height values were taken as a basis. Because the transient signals obtained in the trap studies were very sharp, peak area values were not consistent with increasing concentrations. For this reason, instead of the peak area, peak height values were used. Experimental findings indicated that peak height values exhibited a consistent correlation with increasing concentrations. By using 90 s trapping time, LOD and LOQ values for the developed trap method were determined to be 3.3 and 11.0 ng L^–1^, respectively. While the RSD% was 2.3%, the working linear range and the correlation coefficient (*R*^2^) were obtained as 0.01–10.0 μg L^–1^ and 0.9978, respectively. Enhancement in sensitivity using the ratio of characteristic concentration (*C*_0_) values is 32.5, while this value is 691.8 and 21.3 ng L^–1^ for the GFAAS and trap methods, respectively.

### 3.5 Interference Studies

In the present work, the impact of every interferent ion was studied by preparing standards with Pb/interferent (w/w) ratios of 1:0.1, 1:1, 1:10, and 1:100. Interference studies were carried out using 2.5 μg L^–1^ Pb under optimum conditions previously determined. The trapping time was 90 s and the sample volume was 6.83 mL. Since the hydride-forming ions are in the same matrix as an analyte, competition may occur between the analyte and the interferent ion on the trap surface and this may cause a decrease in the trapping efficiency of the analyte. Results are given in Table 4. The presence of 100-fold Hg, Bi, and Sb resulted in a 12.4, 9.7, and 10.7% increase in the signal, respectively. Hg, Bi, and Sb showed no significant interference effect at the other concentrations. Se and Sn did not show a severe interference effect when they were 0.1-, 1.0-, and 10-fold, but when the interferent amounts were 100-fold of the analyte, the signal decreased by 8.7 and 11.4%, respectively. When the interference/analyte ratio for As was 0.1 and 1.0, no significant change in the signal was observed. However, when the interference/analyte ratio was 10 and 100, there was a decrease in the signals. The signals showed a decrease of 8.3 and 13.2%, respectively.

**Table 4:** Investigating the Effects of Other Hydride-Forming Ions on Pb Determination^a^

	recoveries (%) in the presence of an interferent concentration^b^ (μg L^–1^)			
interferent	0.1	1.0	10.0	100.0
Hg	100.2 ± 1.2	100.5 ± 1.5	108.4 ± 1.8	112.4 ± 1.6
Se	98.6 ± 2.6	98.1 ± 2.3	96.5 ± 1.9	91.3 ± 3.4
Bi	99.5 ± 2.3	101.6 ± 2.4	103.6 ± 3.8	109.7 ± 3.6
Sn	100.8 ± 2.5	97.9 ± 3.3	92.5 ± 4.3	88.6 ± 2.4
Sb	99.6 ± 3.2	100.9 ± 1.7	105.8 ± 2.6	110.7 ± 3.5
As	99.4 ± 2.9	99.1 ± 2.5	91.7 ± 3.1	86.8 ± 2.8

a Results are given as average ± standard deviation (*n* = 3).

b Analyte concentration of 2.5 μg L^–1^.

### 3.6 Accuracy Evaluation

The accuracy of the developed trap method was evaluated by analysis of NIST 1640a and DOLT:5 certified reference materials. A calibration plot was generated externally to measure the Pb content in certified reference materials under optimum experimental conditions, and no standard addition technique was required. The obtained results were in good agreement with the certified values at a 95% confidence level as shown in Table 5.

**Table 5:** Accuracy Evaluation of the Proposed Method Using the Certified Reference Materials^a^

standard reference material	certified value	found value
NIST 1640a (μg L^–1^)	12.101 ± 0.050	12.134 ± 0.028
DOLT:5 (mg kg^–1^)	0.162 ± 0.032	0.178 ± 0.015

a Results are given as average ± standard deviation (*n* = 3).

### 3.7 Analysis of Drinking Water and Fish Tissue Samples

To test the applicability of the proposed trap method, the Pb concentrations in some drinking water and fish tissue samples were analyzed. However, the concentration of Pb in drinking water samples could not be determined under optimal experimental conditions. As an alternative, the drinking water samples were spiked with Pb standard solution at concentrations of 1.5 and 2.0 μg L^–1^, which were within the linear working range of the trap method. For the drinking water samples, the performance characteristics of the method are summarized in Table 6. The *t* test is one of the most widely known statistical tests. To compare the results obtained, a *t* test was performed at a 95% confidence level. The *t*_calculated_ values for the drinking water samples were 3.038, 1.644, 2.422, and 1.638, respectively, all of which were less than the *t*_critical_ value of 4.303. Since *t*_calculated_ values are less than the *t*_critical_ value, the null hypothesis is accepted at the 95% confidence level. It was observed that there was no significant difference between the added and measured concentrations in water samples and it was concluded that there was no systematic error. This also shows that the developed method can be applied to the analysis of similar samples.

**Table 6:** Application of the Proposed Method for Pb Determination in Spiked Water Samples

sample	proposed method (μg L^–1^)	added (μg L^–1^)	found (μg L^–1^)	an error of measurement (μg L^–1^)	an error of measurement (%)	precision (sd)^a^ (μg L^–1^)	precision (RSD%)^b^	MU^c^	accuracy (recovery) (%)
drinking water 1	<LOD	1.5	1.554	0.054	4	0.031	1.98	2.29	104
drinking water 2	<LOD	1.5	1.538	0.038	3	0.040	2.58	2.98	103
drinking water 3	<LOD	2.0	2.096	0.096	5	0.069	3.29	3.79	105
drinking water 4	<LOD	2.0	2.075	0.075	4	0.080	3.84	4.43	104

a standard deviation.

b relative standard deviation.

c MU: measurement uncertainty, *k* = 2, 95% confidence level.

The mean Pb concentrations in different tissues of the sampled fish species, red mullet and common pandora, are presented in Table 7. In both fish species, the highest Pb concentration was detected in the liver tissues. The second highest Pb concentrations were obtained in the gill tissues. The highest Pb concentration allowed in fish tissue was determined by WHO, European Union, and Turkish Food Codex as 0.5, 0.1, and 1.0 μg L^–1^, respectively.^32^ It is concluded that the results obtained were below the values determined by these institutions.

**Table 7:** Concentrations of Pb in Fish Tissue Samples^a^

sample	muscle (μg kg^–1^)	liver (μg kg^–1^)	gill (μg kg^–1^)
red mullet 1	3.5 ± 0.5	14.2 ± 0.6	8.1 ± 0.4
red mullet 2	2.6 ± 0.3	12.5 ± 0.5	6.8 ± 0.3
red mullet 3	2.9 ± 0.3	12.9 ± 0.3	7.2 ± 0.3
common pandora 1	2.2 ± 0.4	10.8 ± 0.3	5.9 ± 0.5
common pandora 2	3.6 ± 0.6	15.3 ± 0.5	9.2 ± 0.4
common pandora 3	4.1 ± 0.6	18.9 ± 0.7	10.5 ± 0.7

a Results are given as average ± standard deviation (*n* = 3).

### 3.8 Comparison of the Proposed Trap Method with Literature Methods

Table 8 compares the performance of the developed Pt-coated W-coil HGAAS method with some other plasma-based and trap methods for Pb determination. The proposed method showed superior precision, with significantly lower RSD% values than those reported in previous studies. A compact quartz tube atom trap method, proposed by Kratzer,^16^ was employed to trap plumbane with a very short preconcentration time of 30 s and achieved 100% preconcentration efficiency, with a detection limit of 210 ng L^–1^. Although this study showed a better detection limit than Kratzer’s study, the preconcentration time in this study (90 s) was much longer. This study achieved a better detection limit for Pb determination than some solid-phase extraction and coated/uncoated slotted quartz tube atom trap methods combined with AAS.^25,33−35^ The comparison revealed that the detection limit obtained with the developed trap method was better than some plasma-based methods.^12,13,35^ According to the results, the present trap method was very sensitive, rapid, and repeatable and it can be used for the ultratrace determination of Pb in environmental and food samples.

**Table 8:** Comparison of the Analytical Features with Reported Earlier Methods

technique	LOD (ng L^–1^)	RSD (%)	references
In-atomizer trapping HGAAS	210.0	<6.0	(16)
SI-SD-μSPE coupled with the AAS	1590	2.4	(32)
SPME-FAAS	330	4.9	(33)
T-SQT-AT-FAAS method	600		(25)
Ta-coated SQT-AT-FAAS	150	3.5	(34)
SPE-ICPOES	73	1.2	(13)
HG-ICPMS	8.0	<6.3	(12)
PVG-ICPMS	3.6	4.4	(36)
HGAFS	2.8	4.4	(14)
SPME-PD-OES	3.0	4.5	(37)
Pt-coated W-coil trap HGAAS	3.3	2.3	this study

## 4 Conclusions

A novel trap method based on HGAAS with in situ preconcentration of Pb hydride species was developed. The Pt-coated W-coil trap is portable, and despite using the same coil in at least 500 burning cycles throughout all experimental studies, no decrease in sensitivity was observed. The *C*_0_ of the developed trap method was indicating a 32.5-fold enhancement in sensitivity compared to GFAAS. Even employing only 90 s of preconcentration time, LOQs comparable to the best ones ever reached for in situ trapping in HGAAS were observed. The detection limit of the obtained trap method was achieved comparable to ICPMS and inductively coupled plasma optical emission spectrometry (ICPOES). It should be emphasized that the overall analytical system is very economical. For researchers who do not have access to costly instruments like ICPMS, ICPOES, and AFS, the developed trap method offers a practical alternative for the determination of ultratrace Pb levels in environmental and food samples. Furthermore, the proposed method successfully eliminates potential interferences, making it a reliable and practical option for analysts. The effectiveness of the developed trap method was demonstrated by successfully analyzing drinking water and various fish tissue samples. The achieved sensitivity is sufficient to overcome several analytical difficulties in accurately determining Pb levels in the fields of health and food, and the environment.
